# The top 100 most cited articles in the last two decades of atopic dermatitis: A bibliometric analysis

**DOI:** 10.3389/fimmu.2022.949665

**Published:** 2022-11-30

**Authors:** Lishan Zhang, Yibo Hou, Jinlyu Sun, Yueping Zeng

**Affiliations:** ^1^ Department of Allergy, State Key Laboratory of Complex Severe and Rare Diseases, Peking Union Medical College Hospital, Chinese Academy of Medical Sciences and Peking Union Medical College, Beijing, China; ^2^ Allergy Department, Beijing Key Laboratory of Precision Medicine for Diagnosis and Treatment of Allergic Diseases, National Clinical Research Center for Dermatologic and Immunologic Diseases, Peking Union Medical College Hospital, Chinese Academy of Medical Sciences and Peking Union Medical College, Beijing, China; ^3^ Department of Dermatology, State Key Laboratory of Complex Severe and Rare Diseases, Peking Union Medical College Hospital, Chinese Academy of Medical Sciences and Peking Union Medical College, National Clinical Research Center for Dermatologic and Immunologic Diseases, Beijing, China

**Keywords:** atopic dermatitis, bibliometric analysis, pathogenesis, treatment, eczema

## Abstract

**Background:**

Atopic dermatitis (AD) is the leading cause of skin-related disease burden worldwide, affecting a large percentage of the population. Bibliometrics is the statistical analysis of academic literature in a certain field.

**Objectives:**

We aimed to perform the latest bibliometric analysis of atopic dermatitis literature.

**Methods:**

We searched the Web of Science database on 29th Nov 2021. We used the keywords “atopic dermatitis,” “atopic eczema,” and “eczema” for our search. All items published between 2001 and 2021 were included. The top 100 most cited articles were identified and analyzed.

**Results:**

Our study provided a detailed bibliometric analysis of the top 100 most cited articles on atopic dermatitis. These articles were published between 2002 and 2019 and were from 15 different countries, mostly in the USA and Germany. Most articles have focused on the pathogenesis and treatment of AD. The Journal of Allergy and Clinical Immunology made the greatest contribution to the top 100 list, with 28 articles. The most cited article originated from Lancet. The highest number of citations was seen in 2006, with 9220 citations, while the highest number of publications was seen in 2006 with 12 publications.

**Conclusions:**

Our study aims to provide physicians and researchers with a historical perspective for the scientific progress of atopic dermatitis, and help clinicians effectively obtain useful articles that have a significant impact on the field of atopic dermatitis.

## Introduction

Atopic dermatitis (AD) is a chronic relapsing inflammatory skin disease, which is characterized by severe itching, dryness, and erythematous rashes (1). AD may seriously affect the quality of life of patients and their families due to intense pruritus, sleeping loss, psychosocial impact, and substantial economic burden caused by costs of medical treatment (2, 3). The prevalence of AD is approximately 20% among children and it ranges between 7% and 14% among adults (4).

The bibliometric study represents an important study type showing the main research focuses or hotspots and trend topics in a given field (5). It could provide a systematic description and visual representation of the structure of a particular field for physicians and researchers. So far, many dermatologists have published the most cited articles in the form of bibliometric analysis on topics such as hidradenitis suppurativa, rosacea, and psoriasis (6–8).

The purpose of our study was to analyze the top 100 most cited AD articles published in the biomedical literature during the last 20 years and pave the way for further investigations for AD.

## Methods

### Search strategy

We have compared the pros and cons for each of them based on AlRyalat et al’s study and we finally chose the Web of Science (WOS) core collection for our bibliometric analysis (9). An important reason for this choice is the availability of a precise and comprehensive description of highly cited papers and hot papers. This feature is not available in other databases. We searched the WOS core database (accessed: 2021-11-29) using the topic “eczema”, “atopic dermatitis”, and “atopic eczema” between 2002 and 2021. There were no limitations on document types or languages. The results were sorted in descending order according to the number of citations cited in the WOS. Two researchers (Lishan Zhang and Yibo Hou) independently screened the abstract to obtain qualified articles. Studies on AD or studies including AD as the main part were included in our analysis scope. Articles not relevant to AD were excluded from our analysis. Under the guidance of senior experts (Yueping Zeng and Jinlyu Sun), two researchers finally reached an agreement on the top 100 most cited articles (T100) list.

### Data extraction and conversion

Data including titles, authors, countries, institutes, year of publication, journals of publication, article types, impact factors, and total citations were collected from the WOS core database to excel. The Countries and institutes were recorded based on the information of the first corresponding author. The types of articles were categorized by original research, review article, clinical trial, clinical study, and comment. Journal impact factors were derived from 2020 Journal Citation Reports. As we all know, a citation is an important metric that shows the quality and influence of an article. However, a certain amount of time should be allowed to pass after the publication of an article for it to reach a higher number of citations. In our study, the average citations per year (ACY) score was used to eliminate the time bias, which provides a fairer comparison for junior academics. ACY = citation times/(2021-publication year+1).

### Bibliometric analysis

Bibliometrix R-package software, an open-source software, was used for conducting quantitative analysis in our study. R-package was developed by Aria and Cuccurullo and written in R language, which can help users without coding skills to perform bibliometric analysis (10). After installing R on our computer, data downloaded from WOS in Plain Text format was uploaded to the Biblioshiny app. The Biblioshiny interface provides us with a set of tools for conducting statistics. Besides, we classified the selected articles into four categories based on the research topic, including pathogenesis, prevention and treatment, epidemiology, and others. “Pathogenesis” includes studies on exploring the origination and development of AD; “prevention and treatment” includes studies on the prevention and treatment of AD; “epidemiology” is the study of diseases in populations, investigating how, when, and why they occur; “others” includes the studies that do not belong to the above categories.

### Ethical statement

This research was designed as a bibliometric analysis of published studies and did not involve any animal experiments or unreported clinical trials of human beings. Thus, approval from an ethics committee was not required.

## Results

A total of 37,937 documents were retrieved from the WOS Core Collection databases between 2002 and 2021. The top 100 most cited articles were listed in **Table 1** and sorted in descending order according to the number of citations. For the study types of the T100 articles on AD, reviews accounted for 37%, followed by basic science researches 27%, clinical trials 21%, clinical studies 10%, comments 1%, and others 4%.

**Table 1:** The top 100 cited articles on atopic dermatitis.

Rank	Article	Citations	ACY*	Topic
1	Asher M I, Montefort S, Bjorksten B, et al. Worldwide time trends in the prevalence of symptoms of asthma, allergic rhinoconjunctivitis, and eczema in childhood: ISAAC Phases One and Three repeat multicountry cross-sectional surveys. Lancet, 2006,368(9537):733-743.	2814	187.6	Epidemiology
2	Compton J G, Munro C S, Palmer C N A, et al. Common loss-of-function variants of the epidermal barrier protein filaggrin are a major predisposing factor for atopic dermatitis. Nat Genet, 2006,38(4):441-446.	1939	129.27	Pathogenesis
3	Soumelis V, Reche P A, Kanzler H. Human epithelial cells trigger dendritic cell-mediated allergic inflammation by producing TSLP. Nat Immunol, 2002,3(7):673-80.	1504	79.16	Pathogenesis
4	Ong P Y, Ohtake T, Brandt C, et al. Endogenous antimicrobial peptides and skin infections in atopic dermatitis. N Engl J Med, 2002,347(15):1151-1160.	1403	73.84	Pathogenesis
5	Bieber T. Atopic dermatitis. N Engl J Med, 2008,358(14):1483-1494.	1385	106.54	Pathogenesis
6	Galli S J, Tsai M, Piliponsky A M. The development of allergic inflammation. Nature, 2008,454(7203):445-454.	1159	89.15	Pathogenesis
7	Leung D Y, Boguniewicz M, Howell M D, et al. New insights into atopic dermatitis. J Clin Invest, 2004,113(5):651-657.	1078	63.41	Pathogenesis
8	Leung D Y, Bieber T. Atopic dermatitis. Lancet, 2003,361(9352):151-160.	1044	58	Pathogenesis
9	Weidinger S, Novak N. Atopic dermatitis. Lancet, 2016,387(10023):1109-1122.	907	181.4	Pathogenesis
10	Kong H, Oh J, Deming C. Temporal shifts in the skin microbiome associated with disease flares and treatment in children with atopic dermatitis. Genome Res, 2012,22(5):850-9.	899	99.89	Pathogenesis
11	Kalliomaki M, Salminen S, Poussa T, et al. Probiotics and prevention of atopic disease: 4-year follow-up of a randomised placebo-controlled trial. Lancet, 2003,361(9372):1869-1871.	896	49.78	Prevention and treatment
12	Simpson E L, Bieber T, Guttman-Yassky E, et al. Two Phase 3 Trials of Dupilumab versus Placebo in Atopic Dermatitis. N Engl J Med, 2016,375(24):2335-2348.	818	163.6	Prevention and treatment
13	Nestle F O, Di Meglio P, Qin J Z, et al. Skin immune sentinels in health and disease. Nat Rev Immunol, 2009,9(10):679-691.	779	64.92	Pathogenesis
14	Liew F Y, Pitman N I, McInnes I B. Disease-associated functions of IL-33: the new kid in the IL-1 family. Nat Rev Immunol, 2010,10(2):103-110.	771	70.09	Pathogenesis
15	Trifari S, Kaplan C D, Tran E H, et al. Identification of a human helper T cell population that has abundant production of interleukin 22 and is distinct from T(H)-17, T(H)1 and T(H)2 cells. Nat Immunol, 2009,10(8):864-871.	768	64	Pathogenesis
16	Beck L A, Thaci D, Hamilton J D. Dupilumab treatment in adults with moderate-to-severe atopic dermatitis. N Engl J Med, 2014,371(2):130-9.	760	108.57	Prevention and treatment
17	Greer F R, Sicherer S H, Burks A W. The Effects of Early Nutritional Interventions on the Development of Atopic Disease in Infants and Children: The Role of Maternal Dietary Restriction, Breastfeeding, Hydrolyzed Formulas, and Timing of Introduction of Allergenic Complementary Foods. Pediatrics, 2019,143(4): e20190281.	741	370.5	Prevention and treatment
18	Irvine A D, McLean W H, Leung D Y. Filaggrin mutations associated with skin and allergic diseases. N Engl J Med, 2011,365(14):1315-1327.	700	70	Pathogenesis
19	Madison K C. Barrier function of the skin: “la raison d’être” of the epidermis. J Invest Dermatol, 2003,121(2):231-41.	681	37.83	Pathogenesis
20	Boguniewicz M, Leung D Y. Atopic dermatitis: a disease of altered skin barrier and immune dysregulation. Immunol Rev, 2011,242(1):233-246.	676	67.6	Pathogenesis
21	Okada H, Kuhn C, Feillet H. The ‘hygiene hypothesis’ for autoimmune and allergic diseases: an update. Clin Exp Immunol, 2010,160(1):1-9.	663	60.27	Pathogenesis
22	Dillon S R, Sprecher C, Hammond A, et al. Interleukin 31, a cytokine produced by activated T cells, induces dermatitis in mice. Nat Immunol, 2004,5(7):752-760.	653	38.41	Pathogenesis
23	Ikoma A, Steinhoff M, Stander S, et al. The neurobiology of itch. Nat Rev Neurosci, 2006,7(7):535-547.	634	42.27	Pathogenesis
24	Eichenfield L F, Tom W L, Berger T G, et al. Guidelines of care for the management of atopic dermatitis: section 2. Management and treatment of atopic dermatitis with topical therapies. J Am Acad Dermatol, 2014,71(1):116-132.	629	89.86	Prevention and treatment
25	Sonkoly E, Muller A, Lauerma A I. IL-31: a new link between T cells and pruritus in atopic skin inflammation. J Allergy Clin Immunol, 2006,117(2):411-7.	610	40.67	Pathogenesis
26	Hay R J, Johns N E, Williams H C. The global burden of skin disease in 2010: an analysis of the prevalence and impact of skin conditions. J Invest Dermatol 2014,134(6):1527-1534.	597	85.29	Epidemiology
27	Penders J, Thijs C, van den Brandt P A, et al. Gut microbiota composition and development of atopic manifestations in infancy: the KOALA Birth Cohort Study. Gut, 2007,56(5):661-667.	570	40.71	Epidemiology
28	Nomura I, Goleva E, Howell M D, et al. Cytokine milieu of atopic dermatitis, as compared to psoriasis, skin prevents induction of innate immune response genes. J Immunol, 2003,171(6):3262-3269.	568	31.56	Pathogenesis
29	Salimi M, Barlow J L, Saunders S P. A role for IL-25 and IL-33-driven type-2 innate lymphoid cells in atopic dermatitis. J Exp Med, 2013,210(13):2939-50.	561	70.13	Pathogenesis
30	Smits H, Engering A, van der Kleij D, et al. Selective probiotic bacteria induce IL-10-producing regulatory T cells *in vitro* by modulating dendritic cell function through dendritic cell-specific intercellular adhesion molecule 3-grabbing nonintegrin. J Allergy Clin Immunol, 2005,115(6):1260-1267.	549	34.31	Pathogenesis
31	Allakhverdi Z, Comeau M R, Jessup H K, et al. Thymic stromal lymphopoietin is released by human epithelial cells in response to microbes, trauma, or inflammation and potently activates mast cells. J Exp Med, 2007,204(2):253-258.	539	38.5	Pathogenesis
32	Eichenfield L F, Tom W L, Chamlin S L, et al. Guidelines of care for the management of atopic dermatitis: section 1. Diagnosis and assessment of atopic dermatitis. J Am Acad Dermatol, 2014,70(2):338-351.	536	67	Prevention and treatment
33	Illi S, von Mutius E, Lau S, et al. The natural course of atopic dermatitis from birth to age 7 years and the association with asthma. J Allergy Clin Immunol, 2004,113(5):925-931.	535	31.47	Epidemiology
34	Kukkonen K, Savilahti E, Haahtela Tari. Probiotics and prebiotic galacto-oligosaccharides in the prevention of allergic diseases: a randomized, double-blind, placebo-controlled trial. J Allergy Clin Immunol, 2007,119(1):192-8.	512	34.13	Prevention and treatment
35	Sun Y G, Chen Z F.A gastrin-releasing peptide receptor mediates the itch sensation in the spinal cord. Nature, 2007,448(7154):700-3.	506	36.14	Pathogenesis
36	Wilson S R, The L, Batia L M, et al. The epithelial cell-derived atopic dermatitis cytokine TSLP activates neurons to induce itch. Cell, 2013,155(2):285-295.	502	62.75	Pathogenesis
37	Mendell M J, Mirer A G, Cheung K, et al. Respiratory and allergic health effects of dampness, mold, and dampness-related agents: a review of the epidemiologic evidence. Environ Health Perspect, 2011,119(6):748-756.	502	50.2	Epidemiology
38	Wang Y H, Angkasekwinai P, Lu N, et al. IL-25 augments type 2 immune responses by enhancing the expansion and functions of TSLP-DC-activated Th2 memory cells. J Exp Med, 2007,204(8):1837-1847.	501	35.79	Pathogenesis
39	Odhiambo J A, Williams H C, Clayton T O, et al.Global variations in prevalence of eczema symptoms in children from ISAAC Phase Three. J Allergy Clin Immunol, 2009,124(6):1251-8.e23.	500	41.67	Epidemiology
40	Howell M D, Kim B E, Gao P, et al. Cytokine modulation of atopic dermatitis filaggrin skin expression. J Allergy Clin Immunol, 2007,120(1):150-155.	499	35.64	Pathogenesis
41	Abrahamsson T R, Jakobsson H E, Andersson A F, et al. Low diversity of the gut microbiota in infants with atopic eczema. J Allergy Clin Immunol, 2012,129(2):434-440, 440-441.	495	55	Pathogenesis
42	Blauvelt A, de Bruin-Weller M, Gooderham M, et al. Long-term management of moderate-to-severe atopic dermatitis with dupilumab and concomitant topical corticosteroids (LIBERTY AD CHRONOS): a 1-year, randomised, double-blinded, placebo-controlled, phase 3 trial. Lancet, 2017,389(10086):2287-2303.	494	123.5	Prevention and treatment
43	Byrd A L, Belkaid Y, Segre J A. The human skin microbiome. Nat Rev Microbiol, 2018,16(3):143-155.	491	163.67	Pathogenesis
44	Gittler J K, Shemer A, Suarez-Farinas M, et al. Progressive activation of T(H)2/T(H)22 cytokines and selective epidermal proteins characterizes acute and chronic atopic dermatitis. J Allergy Clin Immunol, 2012,130(6):1344-1354.	482	53.56	Pathogenesis
45	Kim B S, Siracusa M C, Saenz S A, et al. TSLP elicits IL-33-independent innate lymphoid cell responses to promote skin inflammation. Sci Transl Med, 2013,5(170):170ra16.	476	59.5	Pathogenesis
46	Shaw T E, Currie G P, Koudelka C W, et al. Eczema prevalence in the United States: data from the 2003 National Survey of Children’s Health. J Invest Dermatol, 2011,131(1):67-73.	471	47.1	Epidemiology
47	Hongbo Y, Thomas C L, Harrison M A, et al. Translating the science of quality of life into practice: What do dermatology life quality index scores mean? J Invest Dermatol, 2005,125(4):659-664.	466	29.13	Others
48	Koga C, Kabashima K, Shiraishi N, et al. Possible pathogenic role of Th17 cells for atopic dermatitis. J Invest Dermatol, 2008,128(11):2625-2630.	464	35.69	Pathogenesis
49	Moro G, Arslanoglu S, Stahl B, et al. A mixture of prebiotic oligosaccharides reduces the incidence of atopic dermatitis during the first six months of age. Arch Dis Child, 2006,91(10):814-819.	463	30.87	Prevention and treatment
50	Sandilands A, Sutherland C, Irvine A D, et al. Filaggrin in the frontline: role in skin barrier function and disease. J Cell Sci, 2009,122(Pt 9):1285-1294.	456	38	Pathogenesis
51	Cork M J, Danby S G, Vasilopoulos Y, et al. Epidermal barrier dysfunction in atopic dermatitis. J Invest Dermatol, 2009,129(8):1892-1908.	455	37.92	Pathogenesis
52	Sidbury R, Davis DM, Cohen DE, et al. Guidelines of care for the management of atopic dermatitis. J Am Acad Dermatol, 2016,315(5):469-479.	454	64.86	Prevention and treatment
53	Nograles K E, Zaba L C, Shemer A, et al. IL-22-producing “T22” T cells account for upregulated IL-22 in atopic dermatitis despite reduced IL-17-producing TH17 T cells. J Allergy Clin Immunol, 2009,123(6):1244-1252.	454	37.83	Pathogenesis
54	Sandilands A, Terron-Kwiatkowski A, Hull P R, et al. Comprehensive analysis of the gene encoding filaggrin uncovers prevalent and rare mutations in ichthyosis vulgaris and atopic eczema. Nat Genet, 2007,39(5):650-654.	446	31.86	Pathogenesis
55	Weidinger S, Illig T, Baurecht H, et al. Loss-of-function variations within the filaggrin gene predispose for atopic dermatitis with allergic sensitizations. J Allergy Clin Immunol, 2006,118(1):214-219.	446	29.73	Pathogenesis
56	Bisgaard H, Li N, Bonnelykke K, et al. Reduced diversity of the intestinal microbiota during infancy is associated with increased risk of allergic disease at school age. J Allergy Clin Immunol, 2011,128(3):646-52.e1-5.	436	43.6	Epidemiology
57	Steinhoff M, Neisius U, Ikoma A, et al. Proteinase-activated receptor-2 mediates itch: a novel pathway for pruritus in human skin. J Neurosci, 2003,23(15):6176-6180.	436	24.22	Pathogenesis
58	Basra M K, Fenech R, Gatt R M, et al. The Dermatology Life Quality Index 1994-2007: a comprehensive review of validation data and clinical results. Br J Dermatol, 2008,159(5):997-1035.	435	33.46	Others
59	Simpson E L, Chalmers J R, Hanifin J M, et al. Emollient enhancement of the skin barrier from birth offers effective atopic dermatitis prevention. J Allergy Clin Immunol, 2014,134(4):818-823.	432	61.71	Prevention and treatment
60	Yoo J, Omori M, Gyarmati D, et al. Spontaneous atopic dermatitis in mice expressing an inducible thymic stromal lymphopoietin transgene specifically in the skin. J Exp Med, 2005,202(4):541-549.	431	26.93	Pathogenesis
61	Bieber T. Atopic Dermatitis. Ann Dermatol, 2010,22(2):125-137.	427	38.82	Pathogenesis
62	Nakatsuji T, Chen T H, Narala S, et al. Antimicrobials from human skin commensal bacteria protect against Staphylococcus aureus and are deficient in atopic dermatitis. Sci Transl Med, 2017,9(378):eaah4680.	424	106	Others
63	Rosenfeldt V, Benfeldt E, Nielsen S D, et al. Effect of probiotic Lactobacillus strains in children with atopic dermatitis. J Allergy Clin Immunol, 2003,111(2):389-395.	415	23.06	Prevention and treatment
64	Nutten A. Atopic dermatitis: gobal epidemiology and risk factors. Ann Nutr Metab, 2015;66 (Suppl 1):8-16.	408	68	Epidemiology
65	Hammad H, Lambrecht B N. Barrier Epithelial Cells and the Control of Type 2 Immunity. Immunity, 2015,43(1):29-40.	408	68	Pathogenesis
66	Rautava S, Kalliomäki M, Isolauri E. Probiotics during pregnancy and breast-feeding might confer immunomodulatory protection against atopic disease in the infant. J Allergy Clin Immunol, 2002,109(1):119-21.	405	21.32	Prevention and treatment
67	Ziegler S F, Artis D. Sensing the outside world: TSLP regulates barrier immunity. Nat Immunol, 2010,11(4):289-293.	389	35.36	Pathogenesis
68	Homey B, Steinhoff M, Ruzicka T, et al. Cytokines and chemokines orchestrate atopic skin inflammation. J Allergy Clin Immunol, 2006,118(1):178-89	387	25.8	Pathogenesis
69	Taylor A L, Dunstan J A, prescott S L. Probiotic supplementation for the first 6 months of life fails to reduce the risk of atopic dermatitis and increases the risk of allergen sensitization in high-risk children: A randomized controlled trial. J Allergy Clin Immunol, 2007,119(1):184-91.	385	25.367	Prevention and treatment
70	Dunstan J A, Mori T A, Barden A, et al. Fish oil supplementation in pregnancy modifies neonatal allergen-specific immune responses and clinical outcomes in infants at high risk of atopy: a randomized, controlled trial. J Allergy Clin Immunol, 2003,112(6):1178-84.	377	20.94	Prevention and treatment
71	Weidinger S, Beck L A, Bieber T, et al. Atopic dermatitis. Nat Rev Dis Primers, 2018, 21;4(1):1.	375	125	Pathogenesis
72	Arslanoglu S, Moro G E, Schmitt J, et al. Early dietary intervention with a mixture of prebiotic oligosaccharides reduces the incidence of allergic manifestations and infections during the first two years of life. J Nutr, 2008,138(6):1091-5.	373	28.69	Prevention and treatment
73	Ring J, Alomar A, Bieber T, et al. Guidelines for treatment of atopic eczema (atopic dermatitis) part I. J Eur Acad Dermatol Venereol, 2012,26(8):1045-60.	367	40.78	Prevention and treatment
74	Morgenstern V, Zutavern A, Cyrys J, et al. Atopic diseases, allergic sensitization, and exposure to traffic-related air pollution in children. Am J Respir Crit Care Med, 2008,177(12):1331-7.	366	28.15	Epidemiology
75	Akids C A, Akdis M, Bieber T, et al. Diagnosis and treatment of atopic dermatitis in children and adults: European Academy of Allergology and Clinical Immunology/American Academy of Allergy, Asthma and Immunology/PRACTALL Consensus Report. Allergy, 2006,61(8):969-87.	361	24.07	Prevention and treatment
76	Williams H, Stewart A, von Mutius E, et al. Is eczema really on the increase worldwide? J Allergy Clin Immunol, 2008,121(4):947-54. e15.	359	27.62	Epidemiology
77	Cork M J, Robinson D A, Vasilopoulos, et al. New perspectives on epidermal barrier dysfunction in atopic dermatitis: gene-environment interactions. J Allergy Clin Immunol, 2006,118(1):3-21.	353	23.53	Pathogenesis
78	Horimukai K, Morita K, Narita M, et al. Application of moisturizer to neonates prevents development of atopic dermatitis. J Allergy Clin Immunol, 2014,134(4):824-830.e6.	351	50.14	Prevention and treatment
79	Williams H C. atopic dermatitis. N Engl J Med, 2005,352(22):2314-24.	350	21.88	Prevention and treatment
80	Viljanen M, Savilahti E, Haahtela T, et al. Probiotics in the treatment of atopic eczema/dermatitis syndrome in infants: a double-blind placebo-controlled trial. Allergy,2005,60(4):494-500.	349	21.81	Prevention and treatment
81	Gupta R, Sheikh A, Strachan D P, et al. Time trends in allergic disorders in the UK. Thorax, 2007,62(1):91-6.	347	24.79	Epidemiology
82	Cevikbas F, Wang X, Akiyama T, et al. sensory neuron-expressed IL-31 receptor mediates T helper cell-dependent itch: Involvement of TRPV1 and TRPA1. J Allergy Clin Immunol, 2014,133(2):448-60.	345	49.29	Pathogenesis
83	Wollenberg A, Wagner M, Günther S, et al. Plasmacytoid dendritic cells: a new cutaneous dendritic cell subset with distinct role in inflammatory skin diseases. J Invest Dermatol, 2002,119(5):1096-102.	344	18.11	Pathogenesis
84	Abrahamsson T R, Jakobsson T, Böttcher M F, et al. Probiotics in prevention of IgE-associated eczema: a double-blind, randomized, placebo-controlled trial. J Allergy Clin Immunol, 2007,119(5):1174-80.	343	24.5	Prevention and treatment
85	Benedetto A D, Rafaels N M, McGirt L Y, et al. Tight junction defects in patients with atopic dermatitis. J Allergy Clin Immunol, 2011,127(3):773-86.e1-7.	339	33.9	Pathogenesis
86	Thaci D, Simpson E L, Beck L A, et al. Efficacy and safety of dupilumab in adults with moderate-to-severe atopic dermatitis inadequately controlled by topical treatments: a randomised, placebo-controlled, dose-ranging phase 2b trial. Lancet, 2016,387(10013):40-52.	336	67.2	Prevention and treatment
87	Oetjen L K, Mack M R, Feng J, et al. Sensory Neurons Co-opt Classical Immune Signaling Pathways to Mediate Chronic Itch. Cell, 2017,171(1):217-228.e13.	328	82	Pathogenesis
88	Fallon P G, Sasaki T, Sandilands A, et al. A homozygous frameshift mutation in the mouse Flg gene facilitates enhanced percutaneous allergen priming. Nat Genet, 2009,41(5):602-8.	326	27.17	Pathogenesis
89	Jin H, He R, Oyoshi M, et al. Animal Models of Atopic Dermatitis. J Invest Dermatol, 2009,129(1):31-40.	325	27.08	Others
90	Kalliomäki M, Salminen S, Poussa T, et al. Probiotics during the first 7 years of life: a cumulative risk reduction of eczema in a randomized, placebo-controlled trial. J Allergy Clin Immunol, 2007,119(4):1019-21.	325	23.21	Prevention and treatment
91	Carroll C L, Balkrishnan R, Feldman S R, et al. The burden of atopic dermatitis: impact on the patient, family, and society. Pediatr Dermatol, 2005;22(3):192-9.	325	20.31	Pathogenesis
92	Elias P M, Hatano Y, Williams M L. Basis for the barrier abnormality in atopic dermatitis: Outside-inside-outside pathogenic mechanisms. J Allergy Clin Immunol, 2008,121(6):1337-43.	323	24.85	Pathogenesis
93	Devereux G, Seaton A. Diet as a risk factor for atopy and asthma. J Allergy Clin Immunol, 2005,115(6):1109-17.	322	20.13	Epidemiology
94	Silverberg J I, Hanifin J M. Adult eczema prevalence and associations with asthma and other health and demographic factors: a US population-based study. J Allergy Clin Immunol, 2013,132(5):1132-8.	318	39.75	Epidemiology
95	Brown S J, McLean W H I. One remarkable molecule: filaggrin. J Invest Dermatol, 2012,132(3 Pt 2):751-62.	317	35.22	Pathogenesis
96	Segre J A. Epidermal barrier formation and recovery in skin disorders. J Clin Invest, 2006,116(5):1150-8.	316	21.07	Pathogenesis
97	Ruzicka T, Mihara R. Anti-Interleukin-31 Receptor A Antibody for Atopic Dermatitis. N Engl J Med, 2017,376(21):2093.	315	78.75	Prevention and treatment
98	Huang J T, Abrams M, Tlougan B, et al. Treatment of Staphylococcus aureus colonization in atopic dermatitis decreases disease severity. Pediatrics, 2009,123(5):e808-14.	315	26.25	Prevention and treatment
99	Werfel T, Allam J P, Biedermann T, et al. Cellular and molecular immunologic mechanisms in patients with atopic dermatitis. J Allergy Clin Immunol, 2016,138(2):336-49.	312	62.4	Pathogenesis
100	Kim B E, Leung D Y M, Boguniewicz M, et al. Loricrin and involucrin expression is down-regulated by Th2 cytokines through STAT-6. Clin Immunol, 2008,126(3):332-7.	310	23.85	Pathogenesis

ACY*- average citations per year.

### Year of publication

**Figure 1:**
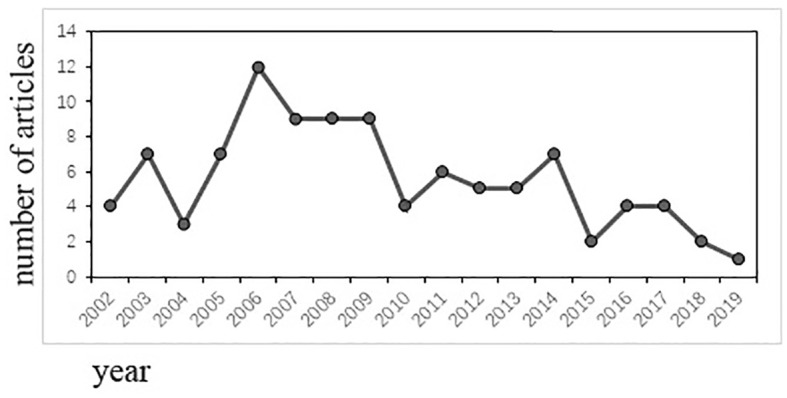
The number of articles published each year.

The top 100 most cited articles were published between 2002 and 2019. **Figure 1** showed the number of articles published during these 18 years. There was a peak in the region of 2006-2009. The largest number of articles published was 12, which occurred in 2006.

### Citations

**Figure 2:**
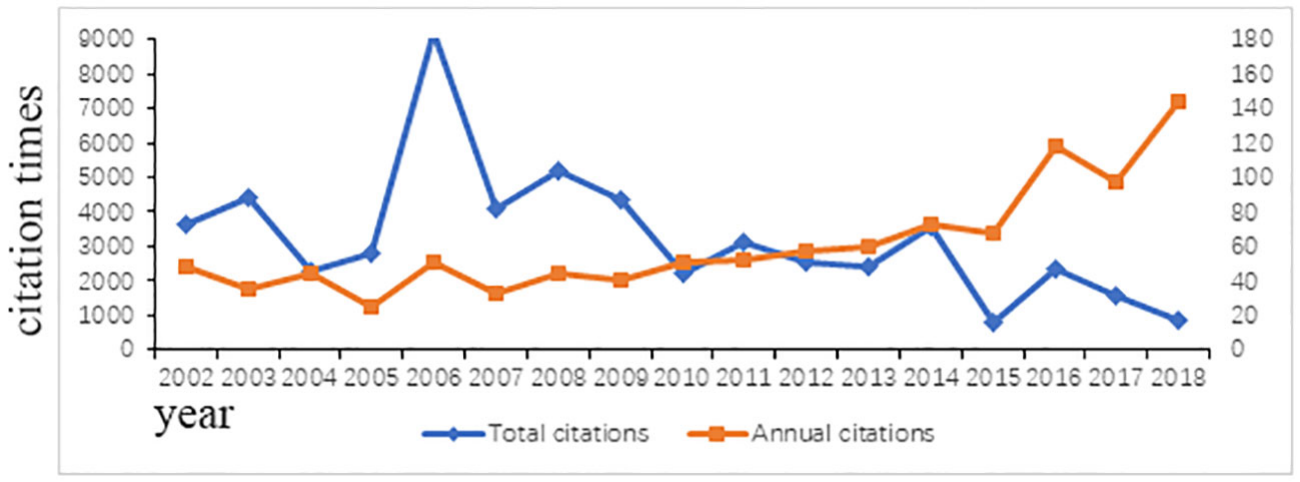
The annual total citations and average annual citations. The orange line represents the average annual citations of the published articles in different year, and the blue line represent the average total citations.

The number of citations ranged from 310 to 2814. We found that the most cited article on AD was a study conducted by Asher et al. with the following title, “Worldwide time trends in the prevalence of symptoms of asthma allergic rhinoconjunctivitis, and eczema in childhood: ISAAC Phases One and Three repeat multicountry cross-sectional surveys,” which was published in *Lancet*, and this article also had the second highest ACY score. The least cited article was “Loricrin and involucrin expression is down-regulated by Th2 cytokines through STAT-6”, which was published in *Clinical Immunology*. The annual total citations and average article citations per year are shown in **Figure 2**. The total number of citations was the highest in 2006, reaching 9220, and the most cited article also appeared in 2006. The average number of annual citations was constantly increasing. Additionally, we found that eight articles received more than 1000 citations, and half of them were published in *Lancet* and *The New England Journal of Medicine*.

### Journal of publication

The 100 articles were published across 37 different journals. *The Journal of Allergy and Clinical Immunology* published the most (n=28), followed by *The Journal of Investigative Dermatology* (n=9), *The New England Journal of Medicine* (n=7), and *Lancet* (n=6). The top 10 journals were listed in **Table 2** with their impact factors and quartile scores. There were 91 articles published in journals in which the “Quartile Score” category was Q1. Of the T100 articles, 74 were published in 24 journals that had an IF>10.

**Table 2:** Top 10 list of journals with published articles.

Journal	Number of articles	Impact factor	Quartile score
Journal of Allergy and Clinical Immunology	28	10.793	1
Journal of Investigative Dermatology	9	8.551	1
New England Journal of Medicine	7	91.245	1
Lancet	6	79.321	1
Nature Immunology	4	25.606	1
Journal of Experimental Medicine	4	14.307	1
Nature Genetics	3	38.33	2
Journal of the American Academy of Dermatology	3	11.527	1
Nature Reviews Immunology	2	53.106	1
Nature	2	49.962	1
Cell	2	41.582	1
Science Translational Medicine	2	17.956	1
Journal Clinical Investigation	2	14.808	1
Allergy	2	13.146	1
Pediatrics	2	7.125	1

### Countries and authors

The first corresponding authors of the 100 articles were from 15 different countries. According to the list, the USA (n=41) was the most contributing country, followed by Germany (n=18) and the United Kingdom (n=17) (**Table 3**). The highest-ranking 10 authors in the T100 cited articles were listed in **Table 4**. We found that Prof. Donald Leung, from the University of Colorado, produced the most top-cited articles on AD (n=12), followed by Prof. Thomas Bieber (n=10).

**Table 3:** Top 10 list of geographic origin.

Country	Number of articles
USA	41
Germany	18
United Kingdom	17
Finland	5
Netherlands	3
Australia	2
Denmark	2
Japan	2
New Zealand	2
Sweden	2

**Table 4:** Top 10 authors with the most published articles.

Corresponding author	Number of articles
Donald Y.M. Leung	12
Thomas Bieber	10
Mark Boguniewicz	8
W.H. Irwin Mclean	8
Eric L. Simpson	8
Jon M. Hanifin	7
Alan D. Irvine	7
Hywel C. Williams	7
Lisa A. Beck	6
Emma Guttman-Yassky	5
Bernhard Homey	5
Martin Steinhoff	5
Stephan Weidinger	5
Andreas Wollenberg	5

### Keywords analysis, co-occurrence network, and trend topics

**Figure 3:**
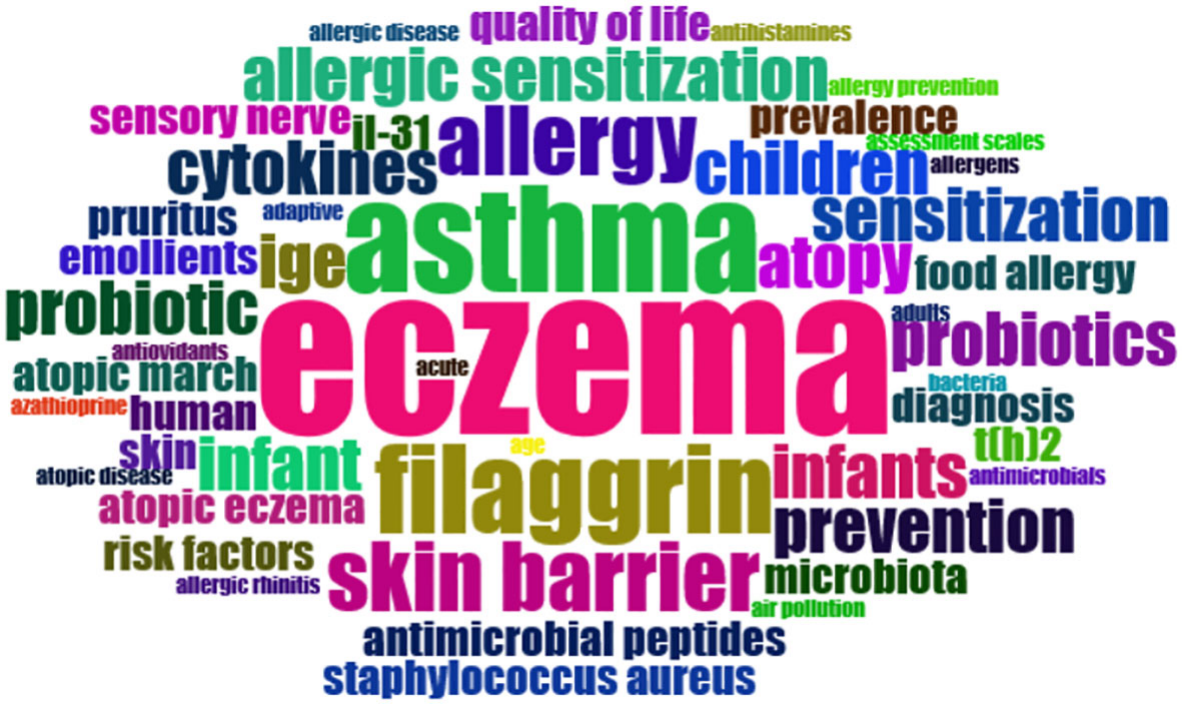
A visualized word cloud of frequently used keywords in the T100 list. The font size represents the number of repetitive keywords.

**Figure 4:**
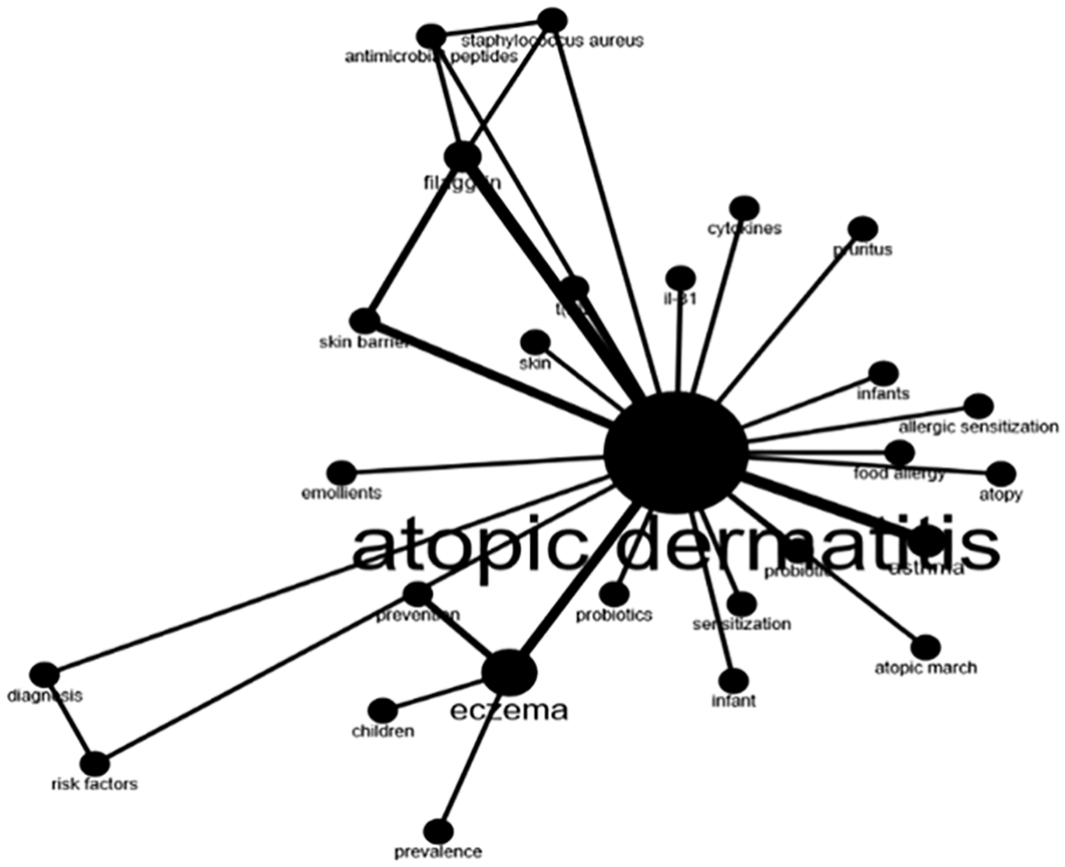
A co-occurrence networks. Thick lines indicate a strong relationship between those keywords. Thin lines represent weak association. Keywords without connecting lines indicate that no relationship has been established.

**Figure 5:**
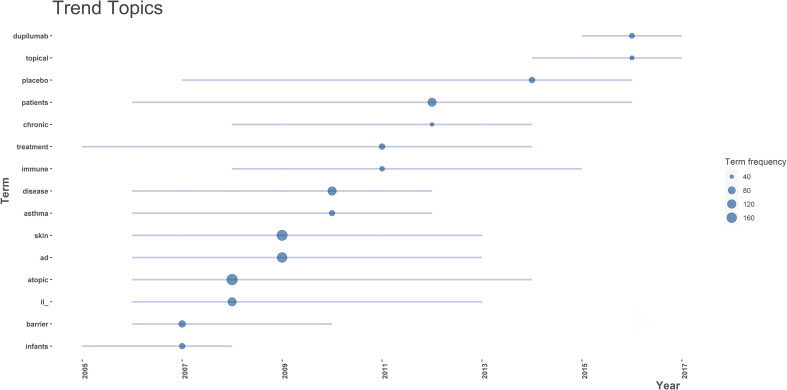
The trend topics analysis presents the hierarchical arrangement of topics in atopic dermatitis (AD) discussed by scholars per year. il, interleukin.

Analysis of authors’ most used keywords is an important tool for exploring hot topics in this field and scholars’ focus. The word cloud in **Figure 3** showed the frequently used keywords were asthma, filaggrin, and skin barrier besides eczema. In addition, we investigated the keywords’ co-occurrence network to reveal the connections between keywords in works of literature (**Figure 4**). Notably, filaggrin played an important role in the co-occurrence network. Trend topics analysis gave further insight into the trending topics in terms of keyword occurrences in AD literature over the years (**Figure 5**). While conducting the analysis, the following parameters were configured. The search field was set to abstract. Word minimum frequency was set to 40 and the number of words per year was set to 2. Cytokines and skin barriers were the most discussed topic between 2006 and 2013. The result also showed that dupilumab (the first biologic agent approved for patients with moderate to severe AD) became a trending topic since 2015.

## Discussion

AD is the most common chronic inflammatory skin disease in many developed countries (11). Recent trends have indicated that the prevalence of AD has increased in industrialized countries in recent decades and poses a significant burden on population health and healthcare settings (12). As we all know, citation analysis can provide comprehensive information about journals, institutions, and authors, which is available for identifying landmark papers and high-impact journals. Although it may not be possible to present a detailed analysis of all 100 top-cited articles, some features could be found. The bibliometric analysis can not only provide a deep insight into the most popular topics of AD in the past but also reveal the trend of AD research (13).

We found that in our T100, 36% of the articles were published less than 10 years prior, while 64% of them were more than 10 years before. However, the total ACY from 2002 to 2010 (ACY=2618) was lower than those from 2011 to 2019 (ACY=3065). It demonstrated that researchers paid more attention to AD in recent years. Although the 100 top-cited articles appeared in a total of 37 different journals, The *Journal of Allergy and Clinical Immunology, Lancet, Cell, Nature*, and *The New England Journal of Medicine*, were the main sources for nearly half of the articles (n=45). We found that core papers related to AD were published in a few top journals and these papers basically covered the overall situations of this field. This finding is consistent with Bradford’s law (14), which estimates the exponentially diminishing returns of extending a search for references in science journals and is commonly applied to determine the core journals in a specific field. Therefore, reading a few top journals could help busy dermatologists to keep updated in this field.

In addition, we noticed that all the articles with a marked influence came from developed countries. However, the latest available data from ISAAC (The International Study of Asthma and Allergies in Childhood), the largest worldwide collaborative research project, showed that AD continued to increase in prevalence specifically in developing countries, such as Latin America or South East Asia (15). Developing countries’ authors have limited involvement mainly due to the imbalance of global health resources between developed countries and developing countries (16).

**Figure 6:**
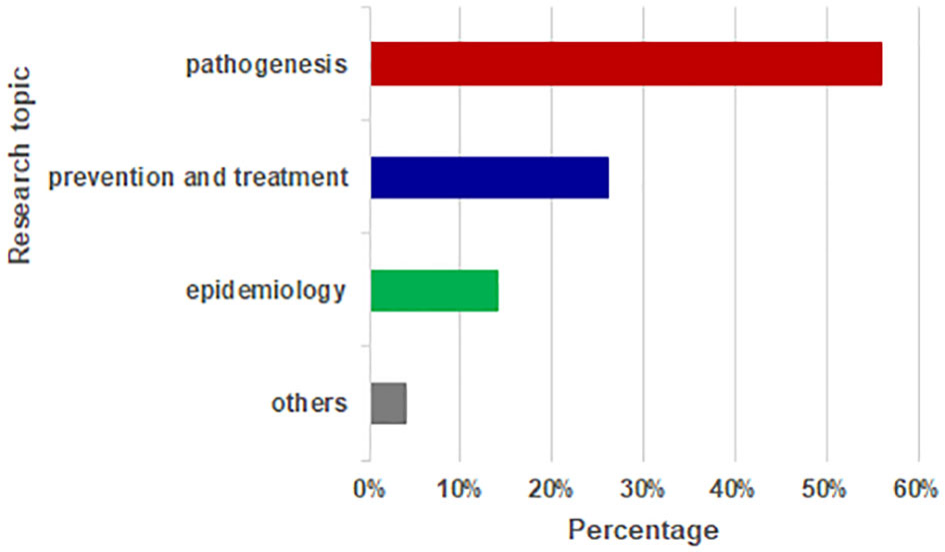
Research topic of published research articles.

The Biblioshiny app screens the themes of the literature according to the frequency of keywords in the selected literature. Sometimes it may not be able to reflect the main content of the article well. Therefore, we summarized the topic of the article manually. Articles were classified into one of the following four domains: pathogenesis, prevention and treatment, epidemiology, and others (**Figure 6** and **Table 1**). Studies related to pathogenesis occupied the most articles (n=56). Extensive research has shed light on the multifaceted pathogenesis of the disease. Although an increasing number of studies have focused on AD, the etiology and pathogenesis of AD remain unclear. The bibliometric analysis shows that AD results from interactions among susceptibility, the environment, food allergy, skin barrier defects, gut microbial alterations, and immune disorders. Of these pathogenesis factors, the most critical one is skin barrier function. The most cited pathogenesis-related article was published in 2006, titled “Common loss-of-function variants of the epidermal barrier protein filaggrin are a major predisposing factor for atopic dermatitis”. This work demonstrated that impaired skin barrier function played a key role in the development of atopic disease. In addition, 15 articles proved that immune cells (Th2 and Th17 cells) and related cytokines (IL-31, IL-25, IL-33, and TSLP) involved in the inflammatory response played an indispensable role in AD. These findings have reflected the direction of research in the last 20 years, and will form an important basis for future research for developing new therapeutic modalities.

Recent developments in the understanding of AD pathogenesis have provided multiple options for treatments. In addition to the conventional therapies (application of moisturizers, topic corticosteroids, and immunosuppressants), targeted drugs including biologics and Janus kinase (JAK) inhibitors have been introduced as new options for AD. Of the T100 list, 26% were related to AD therapy. Sorting the literature in descending order of publication year, we observed that clinical trials on the safety and effectiveness of dupilumab were at the top ten list. The most cited article about dupilumab was a clinical trial published in the *New England Journal of Medicine*, titled “Two Phase 3 Trials of Dupilumab versus Placebo in Atopic Dermatitis”. It is only a 5-year-old article; however, it has 818 citations with an ACY score of 163.6 (rank: fifth). Dupilumab was the first biologic approved for moderate-to-severe AD and has become one of the most promising treatments for AD. Until the emergence of dupilumab, corticosteroids and immunosuppressant agents were the first-line therapies for rapid control of moderate-to-severe AD. Compared with conventional immunosuppressive therapy, dupilumab has good safety and can be used for the long-term treatment of AD (17). However, high cost may be a major consideration for dupilumab. To date, dupilumab has been approved for the remedy of type 2 immune diseases, such as asthma, and chronic rhinosinusitis with nasal polyps. Dupilumab will be used to relieve unmet clinical needs in other type 2 inflammation-related diseases (18). Furthermore, numerous biologics are now under investigation, including anti-IL-31RA monoclonal antibodies (nemolizumab) and anti-IL-13 monoclonal antibodies (tralokinumab and lebrikizumab). Besides, JAK inhibitors represent an emerging treatment option for AD. Abrocitinib, upadacitinib, and baricitinib are three oral JAK inhibitors approved for treating patients with AD, along with topical drugs ruxolitinib and delgocitinib. However, additional real-world studies are necessary to provide valuable data on the response and tolerability of these JAK inhibitors. The Global Burden of Disease has reported that skin diseases continue to be the fourth largest cause of nonfatal disease burden and AD accounts for the highest disease burden among skin diseases (19). Among the T100 list, there were fourteen articles related to the epidemiology of AD. One was a large multicenter cross-sectional survey conducted by Asher et al. and published in the *Lancet*, which has the highest citation number in the T100 list. Measuring the frequency of AD in a population and identifying how the disease frequency may differ over time or among subgroups are important steps in discovering potential causes and determining effective methods for prevention and care.

When we looked at the T100 list, another point of interest also caught our attention: the relationship between AD and intestinal microflora or skin microflora. Evidence suggested that skin and gut microbes might affect the course of AD. Six articles introduced research in this area. The most cited one was published in *Genome Research*, titled “Temporal shifts in the skin microbiome associated with disease flares and treatment in children with atopic dermatitis”. Studies indicated that an imbalance of the intestinal flora might be a factor leading to the deterioration of AD. Scientists have also proposed that the application of probiotics can regulate intestinal health by affecting the quantity and abundance of the intestinal microflora and modulating AD progression. Although several articles have described the efficacy of probiotics in AD, the exact curative effect remains uncertain.

In conclusion, this study identified the top 100 most cited articles on AD and analyzed their bibliometric characteristics, which may facilitate further research. However, there are still some shortcomings and limitations in our study. Firstly, the number of citations used did not exclude self-citations. Secondly, we only used the WOS database to conduct our research. Therefore, the list would be different if other databases were used.

## Data availability statement

The raw data supporting the conclusions of this article will be made available by the authors, without undue reservation.

## Author contributions

YZ, JS contributed to conception and design of the study. LZ, YH performed the statistical analysis and wrote the first draft of the manuscript. All authors contributed to manuscript revision, read, and approved the submitted version.

## Funding

This study was supported by the National High Level Hospital Clinical Research Funding (2022-PUMCH-B-092; Dr. YZ); the National Natural Science Foundation of China (81602765; Dr. YZ), (81971515; Dr. JS).

## Conflict of interest

The authors declare that the research was conducted in the absence of any commercial or financial relationships that could be construed as a potential conflict of interest.

## Publisher’s note

All claims expressed in this article are solely those of the authors and do not necessarily represent those of their affiliated organizations, or those of the publisher, the editors and the reviewers. Any product that may be evaluated in this article, or claim that may be made by its manufacturer, is not guaranteed or endorsed by the publisher.
